# Age, Sex, and Laterality-Based Evaluation of Renal Volume and Surface Area Using CT-Based Three-Dimensional Segmentation

**DOI:** 10.3390/life16081347

**Published:** 2026-08-17

**Authors:** Ayse Gamze Ozcan, Busra Pirinc, Betul Sevindik, Ekrem Solmaz, Mustafa Koplay, Ahmet Kagan Karabulut

**Affiliations:** 1 Department of Anatomy, Faculty of Medicine, Selcuk University, 42130 Konya, Turkey; busrapirinc2016@gmail.com (B.P.); drbetulsevindik@gmail.com (B.S.); drsolmazekrem@gmail.com (E.S.); akkarabulut@yahoo.com (A.K.K.); 2 Department of Radiology, Faculty of Medicine, Selcuk University, 42130 Konya, Turkey; koplaymustafa@hotmail.com

**Keywords:** 3D Slicer, computed tomography, kidney surface area, kidney volume, morphometry

## Abstract

**Background:** The kidneys maintain systemic homeostasis, making renal size assessment essential for evaluating various disorders. Renal volume represents a critical morphometric parameter with distinct clinical significance. This study investigated age-, sex-, and laterality-related differences in renal volume and surface area within an adult population. **Methods:** A total of 582 kidneys from 291 individuals aged 19 to 89 who underwent abdominal CT scans were retrospectively examined. The images were imported into the 3D Slicer software (version 5.6.2) in DICOM format, and automatic segmentation was performed using the Total Segmentator module. The volumes and surface areas of both kidneys were measured and compared according to age and sex groups. **Results:** Kidney volume and surface area values were found to be significantly higher in men than in women (*p* < 0.05). This difference was particularly pronounced in the 30–69 age range. The highest volume values were found in women in the 40–49 age group, while in men, they were found in the 30–59 age group. However, in groups over seventy years old, the gender difference lost its statistical significance. Additionally, the left kidney was found to be significantly larger than the right kidney (*p* < 0.05). As age increases, a gradual decrease in kidney volume and surface area is observed in both sexes. **Conclusions:** The progressive decline in renal metrics and the disappearance of sex-based differences after 70 years align with age-related parenchymal atrophy, nephron loss, and reduced blood flow. These findings support the establishment of data baselines for clinical monitoring and surgical planning.

## 1. Introduction

The kidneys are a pair of organs located retroperitoneally in the body at the T12-L3 level. Due to the liver being located in the abdominal cavity on the right side, the right kidney is positioned slightly lower than the left kidney [1]. The kidneys maintain systemic homeostasis, making renal size assessment essential for evaluating various disorders. Determining kidney size is important in the clinical setting because it can help in diagnosis and determining treatment strategy. Among the various indices used for kidney size, kidney length has traditionally been preferred because it can be easily measured with ultrasonography (USG). However, considering the complexity of kidney shape, kidney length cannot adequately represent kidney volume. It has been shown that ultrasonographic volume calculations based on ellipsoid formulas underestimate kidney volume and have limited reliability due to interobserver differences [2,3]. Kidney volume is a more sensitive size index than kidney length for detecting kidney abnormalities. It is also an excellent predictor of kidney function and correlates well with body indices [4]. With the emergence of more advanced imaging methods such as magnetic resonance imaging (MRI) and computed tomography (CT), it has become possible to measure organ volume. When choosing an imaging method, critical issues include the accuracy, ease, and consistency of the measurement. CT, which generates three-dimensional reconstruction images, is particularly suitable for measuring organ volume [5,6].

In studies using CT, formulas were initially used for volume calculation, but with technological advancements, programs were developed to generate three-dimensional images from the scans. These have made volume calculation considerably easier. With the recent advancements in technology, semi-automatic and automatic segmentation methods have been developed and validated for CT volume measurement of abdominal organs, including volume measurements of the entire [7,8]. One of these programs, 3D Slicer version 5.6.2, (https://www.slicer.org/, accessed on 15 May 2025), is a free and open-source software program. Rather than performing automated segmentation intrinsically, 3D Slicer enables the integration of advanced extension modules. In this study, automatic segmentation was driven by the TotalSegmentator extension module executed within the 3D Slicer platform. It allows for the calculation of organ volumes and various measurements from CT and MRI images and has been found to be highly reliable in comparison studies [9]. 3D Slicer is widely used in calculating the volume and dimensions of kidney stones when they form, calculating the volume of the donor’s kidney in kidney transplants and monitoring the post-transplant process, preparing assistants and students for surgery on preoperative three-dimensional models, monitoring kidney volume in chronic kidney disease, especially autosomal dominant polycystic kidney disease (ADPKD), and monitoring kidney dimensions and glomerular filtration rate (GFR), such as vascular variations [10,11,12,13,14,15]. Given the clinical importance of age- and sex-related variations in renal dimensions, evaluating situations where renal volume fluctuates is critical for monitoring disease progression and clinical follow-up. Numerous radiological imaging techniques and quantitative methodologies have been investigated in the literature to estimate renal volume, with their diagnostic reliability and accuracy extensively documented [16]. Renal dimensions are significantly influenced by physiological variables such as age, sex, and body surface area. However, in the available literature, no study has systematically investigated the age-related morphometric changes of the kidneys across such a broad age range using a sex-specific, decade-based classification approach combined with modern automated segmentation tools. Therefore, renal volume and surface area measurements were performed using the TotalSegmentator module within the 3D Slicer software platform, and the obtained data were compared across age, sex, and laterality groups to analyze potential morphometric variations. By identifying these age- and sex-related differences, we aimed to contribute to a better understanding of normal renal morphometry and to provide objective data that may support clinical decision-making in diagnostic, therapeutic, and surgical processes.

## 2. Materials and Methods

**Figure 1:**
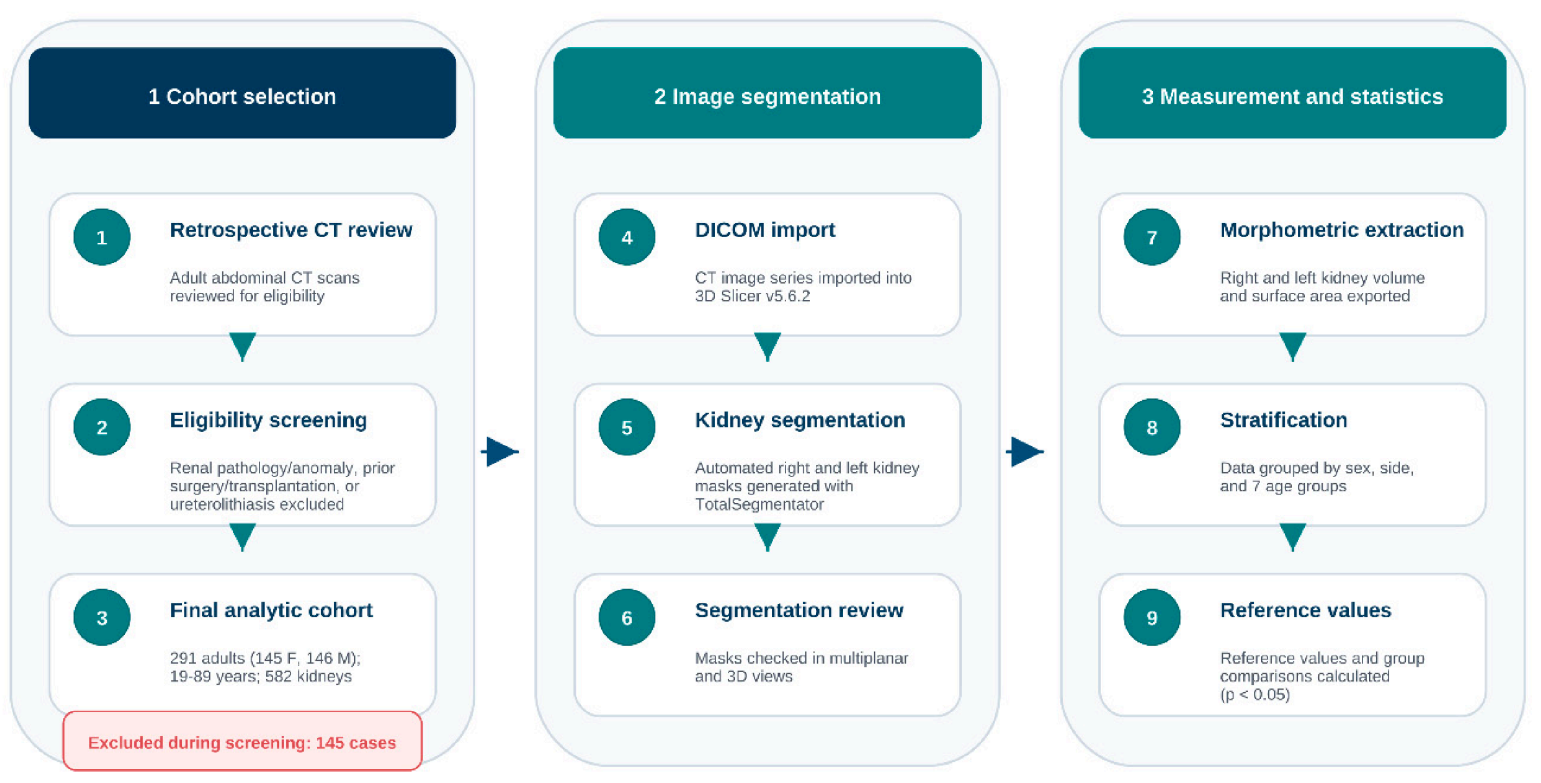
Workflow of the study design and image analysis.

In our study, 436 patients who underwent abdominal CT for various reasons at the Department of Radiology, Faculty of Medicine, Selcuk University, between September 2014 and September 2024, and whose ages ranged from 19 to 89 years, were included. Ethical approval for the study was obtained from the Local Ethics Committee of the Faculty of Medicine, with the approval number 2024/635. A total of 145 individuals were excluded because of radiologically detected conditions that could affect renal morphology, including hydronephrosis, multiple renal cysts, renal artery stenosis, renal tumors, horseshoe kidney, ureteral tumors, congenital renal malformations, previous renal surgery, kidney transplantation, polycystic kidney disease, and ureterolithiasis. Furthermore, the electronic medical records of all participants were reviewed, and those with a documented history of hypertension, diabetes mellitus, chronic kidney disease, or other systemic disorders known to influence renal morphology were excluded. The volume and area of the kidney were measured, and the volumes and areas of the right and left kidneys were compared. Changes based on sex were examined. Additionally, patients were divided into seven groups based on ten-year age ranges (19–29, 30–39, 40–49, 50–59, 60–69, 70–79, 80–89 years), and age-related changes were analyzed (Figure 1).

**Figure 2:**
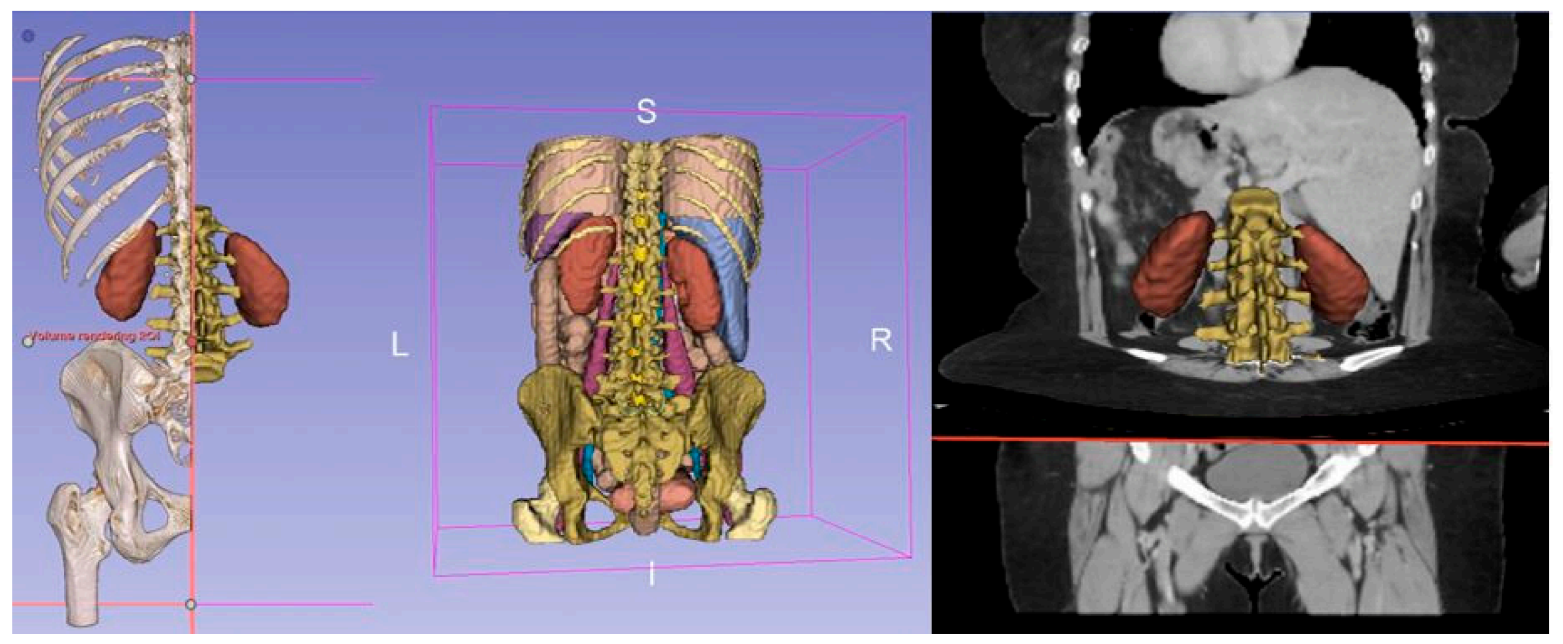
Representative three-dimensional reconstruction of the automatically segmented kidneys generated using the Total Segmentator framework within 3D Slicer. The segmented kidneys are displayed together with the vertebral column to provide anatomical orientation, while the corresponding coronal CT image demonstrates the anatomical localization of the segmented structures before morphometric analysis (L: left, R: right, S: superior).

In the study, patient images were exported in Digital Imaging and Communications in Medicine (DICOM) format, and DICOM data were saved to a personal computer. For the volumetric analysis of the examined structures, 3D Slicer (https://www.slicer.org/, version 5.6.2), a free and open-source software program, was used, and the images loaded into the 3D Slicer program were viewed in three planes (axial, coronal, and sagittal). Suitable images for analysis were selected, and the following steps were followed for the segmentation process [17]. The “Modules” list in the program was accessed, and from there, “Total Segmentator” was selected, and structures were added from the “Add” toolbar. In the total segmentator section, the “Apply” tab was used to assign different colors to the abdominal organs, and then the images were visualized in 3D using the “Show 3D” tab (Figure 2).

**Figure 3:**
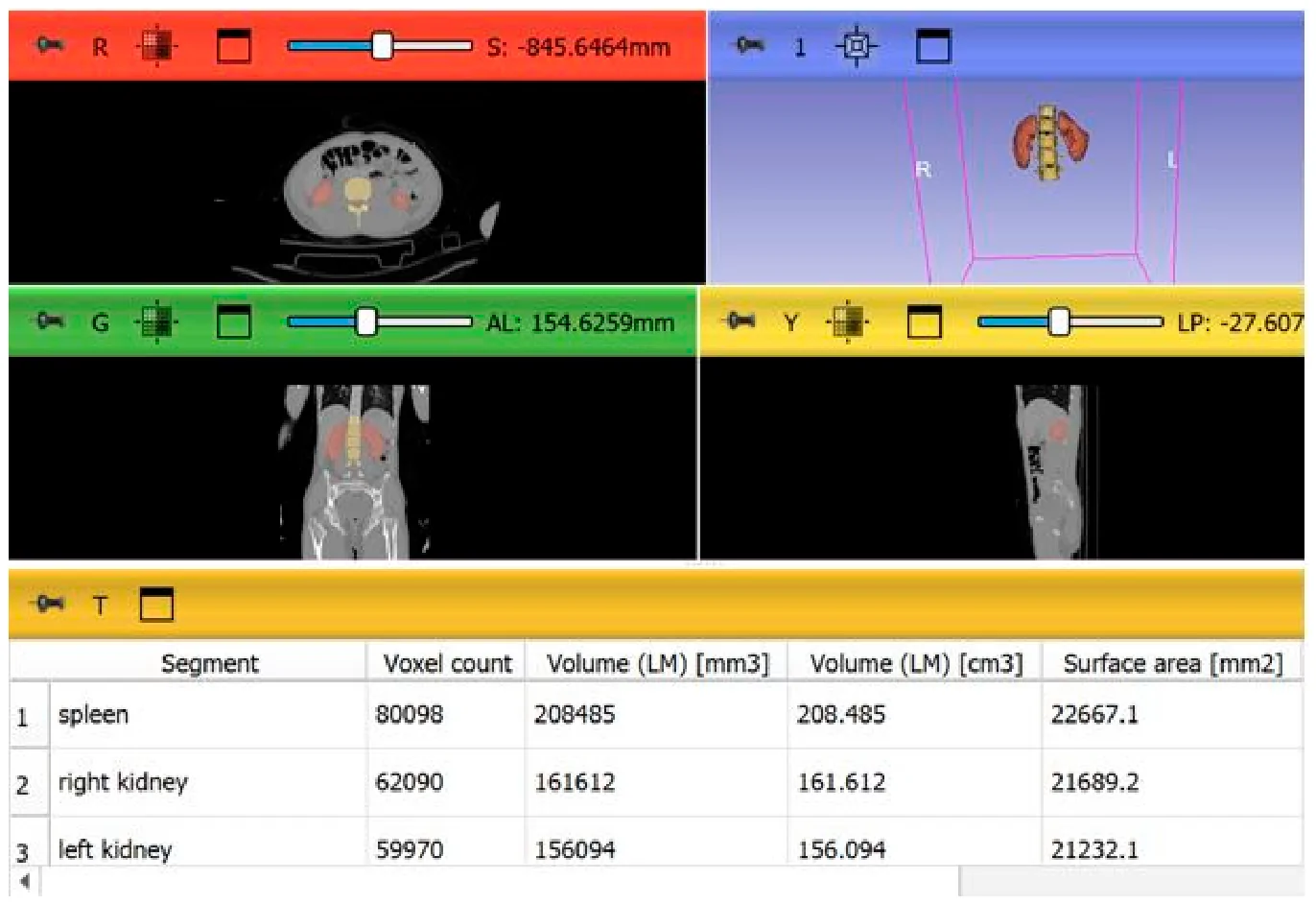
Representative workflow for morphometric analysis in 3D Slicer. Automated kidney segmentations are displayed simultaneously in axial, coronal, sagittal, and three-dimensional views for visual quality assessment. Following segmentation verification, renal volume and surface area were automatically calculated using the Quantification module (L: left, R: right).

As a final step, volume and area measurement reports were obtained from the “Modules” list using the “Quantification” option [18] (Figure 3). All three-dimensional measurements obtained using this method were independently reviewed by two experienced investigators prior to the final analysis. Each investigator performed the measurements independently, and any discrepancies were resolved by consensus following joint evaluation. Interobserver reliability was assessed using the intraclass correlation coefficient (ICC) based on a two-way mixed-effects model with absolute agreement for average measurements. We found excellent reliability as the values were greater than 0.9 (95%) in all parameters.

### 2.1. CT Technique

All CT scans were performed in the supine position using a CT scanner (128 × 2 detector, Somatom Definition Flash, Siemens, Forchheim, Germany). A craniocaudal scan was performed, including the upper abdomen and pelvic region. Following intravenous administration of the non-ionic iodinated contrast agent iohexol (Omnipaque^®^ 300, 300 mgI/mL; GE Healthcare, Cork, Ireland), multiphasic abdominal CT examinations were performed according to the clinical indication. For the purposes of this study, only portal venous phase images (60–70 s after contrast administration) were used for three-dimensional segmentation and morphometric analysis. Contrast medium was administered through a peripheral vein at a rate of 1.8–2.7 mL/s with a dose of 0.8–1.0 mL/kg (maximum 100 mL). Axial images were reconstructed with a slice thickness of 3 mm using a tube voltage of 120 kVp, a pitch of 0.8, and automatic tube current modulation (effective mAs).

### 2.2. Statistical Analysis

The data were evaluated using the IBM SPSS Statistics Standard Concurrent User V 26 statistical software package (IBM Corp., Armonk, NY, USA). Descriptive statistics were presented as the number of units (*n*), percentage (%), mean (X), and standard deviation (SD) values. At the decision stage, if the absolute skewness value is below ±2.0 and the kurtosis value is below 7.0, it is decided that the data were considered normally distributed [19]. Accordingly, the skewness and kurtosis values of the variables used in the study were calculated, and the data were found to be suitable for a normal distribution. Chi-square tests (Pearson chi-square/Fisher’s exact test) were used to evaluate sex across age groups. Measurements of right and left kidney volume in age groups by sex were evaluated using the Paired *t*-test. For paired comparisons, mean paired differences with 95% confidence intervals and Cohen’s dz effect sizes were reported. Comparison of sex by age groups: Independent Samples *t*-test was used for the comparison of sex by age groups, and Analysis of Variance (ANOVA) was used for the comparison of age groups by sex. For multiple comparisons following ANOVA, the Tukey honestly significant difference (HSD) test was applied. Eta-squared (η^2^) values were reported as measures of effect size for the ANOVA results. Mean differences with Tukey-adjusted 95% confidence intervals were reported for statistically significant pairwise comparisons. A *p*-value of <0.05 was considered statistically significant.

## 3. Results

In our study, a total of 582 kidneys from 291 individuals were evaluated, including 145 women (49.8%) and 146 men (50.1%). Individuals aged between 19 to 89 were examined in 7 different age groups, and it was observed that the distribution of age groups by sex was similar (*p* = 0.999) (Table 1). Morphometric findings were evaluated according to sex, side, and age.

### 3.1. Right-Left Kidney Comparison

The mean left kidney volume was significantly greater than the right in the total sample (*p* = 0.001). When all age groups were evaluated together, left kidney volume was also significantly greater in women (*p* = 0.015) and men *p* = 0.028), with small effect sizes. In age-specific analyses, significantly greater left kidney volumes were observed in women aged 19 to 29 (*p* = 0.047) and 50 to 59 (*p* = 0.042), and in men aged 19 to 29 (*p* = 0.005). No statistically significant right–left differences were observed in the remaining age groups (Table 2).

The mean left kidney surface area was significantly greater than the right in the total sample (*p* < 0.001). When all age groups were evaluated together, left kidney surface area was also significantly greater in women (*p* = 0.003) and men (*p* < 0.001). In age-specific analyses, significantly greater left kidney surface areas were observed in men aged 19 to 29 (*p* = 0.009) and 70 to 79 (*p* = 0.010). No statistically significant right–left differences were observed in women or in the remaining male age groups (Table 3).

**Figure 4:**
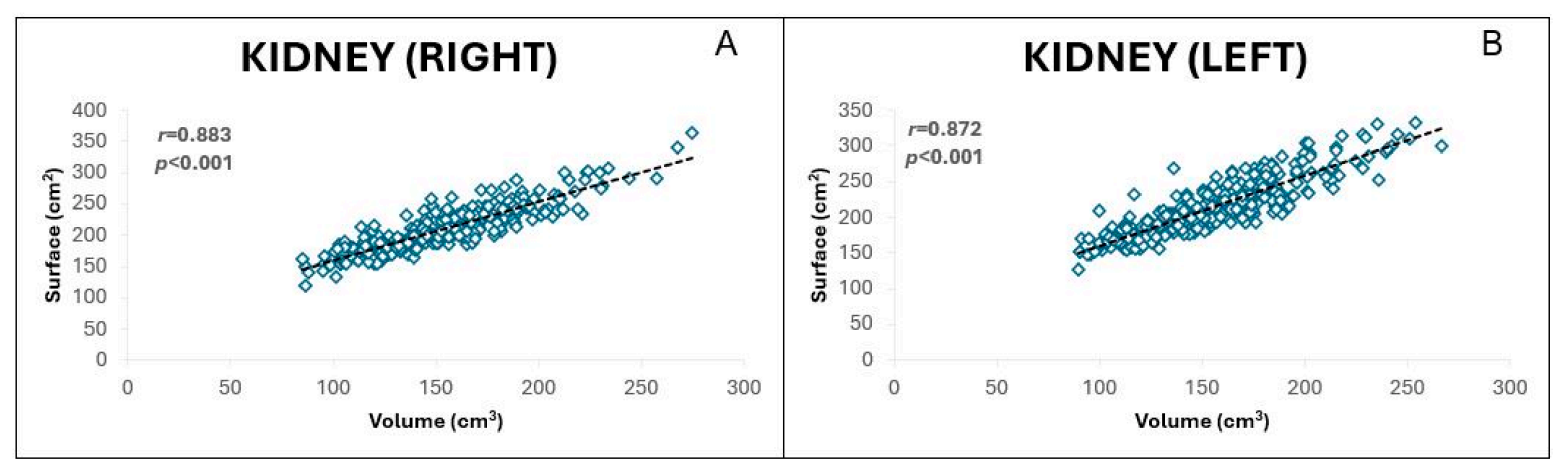
Correlation between renal volume and surface area. (**A**) Right kidney and (**B**) left kidney. Blue diamonds represent individual measurements, and the dashed line indicates the linear regression line. Pearson correlation coefficients were r = 0.883 and r = 0.872, respectively (*p* < 0.001 for both).

There was a statistically significant positive correlation between the area and volume of the right and left kidneys (r = 0.883 *p* < 0.001; r = 0.872 *p* < 0.001) (Figure 4).

### 3.2. Comparison by Age and Sex

Table 4 shows that the average volumes of the right and left kidneys differed statistically significantly by age for women (*p* < 0.05). In women, the volumes of the right and left kidneys were highest in the 40–49 age group and lowest in the 80–89 age group. Tukey HSD analysis showed that right kidney volume in the 80–89-year group was significantly lower than that in the 30–39- and 40–49-year groups, whereas left kidney volume in the 80–89-year group was significantly lower than that in the 30–39-, 40–49-, and 50–59-year groups. The average kidney volume of both sides varied statistically significantly with age in men (*p* < 0.05). In men, the highest values in both kidneys were found in the 30–59 age group, and the lowest values were found in the 70 and over age group. For the right kidney, the volumes in the 30–39-, 40–49-, and 50–59-year groups were significantly higher than those in both the 70–79- and 80–89-year groups. For the left kidney, the volumes in the 30–39-, 40–49-, and 50–59-year groups were significantly higher than that in the 80–89-year group.

The average right and left kidney volumes of participants aged 30 to 69 and 80 to 89 differed significantly by sex (*p* < 0.05). In addition, right kidney volume differed significantly by sex in the 19–29-year group, whereas left kidney volume did not (*p* = 0.069). The average right and left kidney volumes of participants aged 70 to 79 did not show statistically significant sex-based differences (*p* > 0.05). Men had significantly higher right kidney volumes than women in the 19–69- and 80–89-year groups, and significantly higher left kidney volumes in the 30–69- and 80–89-year groups.

One-way ANOVA demonstrated significant age-related differences in right kidney volume in both women (F = 3.394, *p* = 0.004, η^2^ = 0.129) and men (F = 7.178, *p* < 0.001, η^2^ = 0.237). Significant age-related differences were also observed in left kidney volume in women (F = 3.350, *p* = 0.004, η^2^ = 0.127) and men (F = 4.695, *p* < 0.001, η^2^ = 0.169). These effect sizes indicated moderate-to-large age-related differences in women and large differences in men. The mean differences and corresponding Tukey-adjusted 95% confidence intervals for statistically significant pairwise comparisons are presented in Supplementary Table S1.

**Figure 5:**
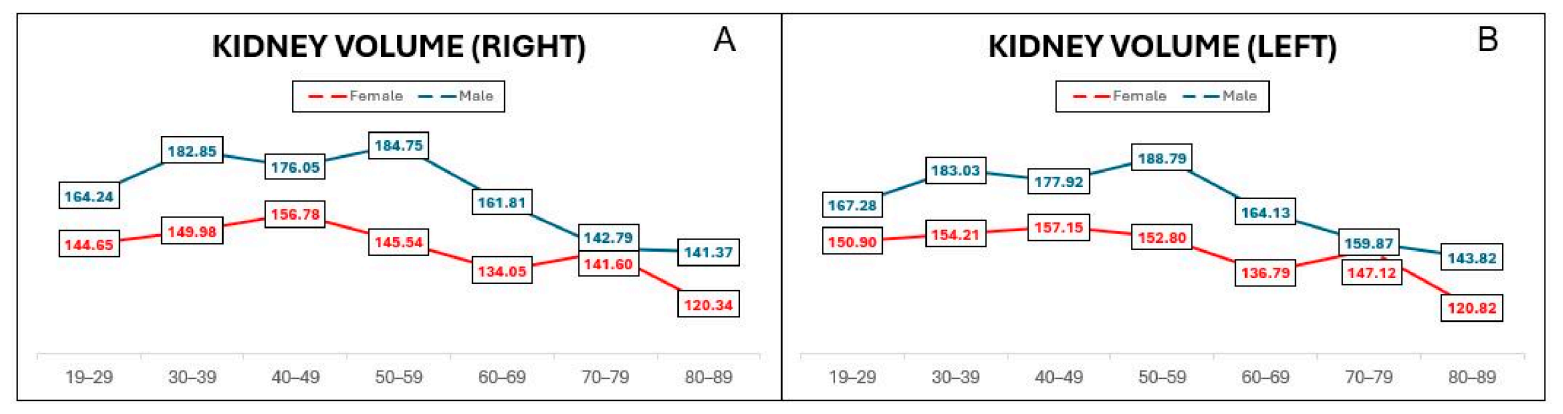
Change in right (**A**) and left (**B**) kidney volume measurement by age group and sex.

As seen in Figure 5, kidney volumes tended to decrease with age in both sexes, and the average kidney volume in men is higher than that in women. No differences were observed between sexes in the 70–79 age group.

The average right and left kidney surface areas differed significantly across age groups in both women and men (*p* < 0.05). In women, right kidney surface area was significantly higher in the 40–49-year group than in all other age groups. For the left kidney, the 40–49-year group had significantly higher surface area values than the 19–29-, 30–39-, 60–69-, 70–79-, and 80–89-year groups, but did not differ significantly from the 50–59-year group. In men, the highest right and left kidney surface area values were observed in the 40–59-year groups. For both kidneys, the 50–59-year group had significantly higher values than the 18–29-, 30–39-, 60–69-, 70–79-, and 80–89-year groups, whereas no significant difference was observed between the 40–49- and 50–59-year groups. The average right and left kidney surface areas differed significantly between sexes in participants aged 19–69 years (*p* < 0.05), whereas no significant sex-based differences were observed in participants aged 70 years or older (*p* > 0.05). Accordingly, men had significantly greater right and left kidney surface areas than women in all age groups between 19 and 69 years (Table 5). The mean differences and corresponding Tukey-adjusted 95% confidence intervals for statistically significant pairwise comparisons are presented in Supplementary Table S2.

**Figure 6:**
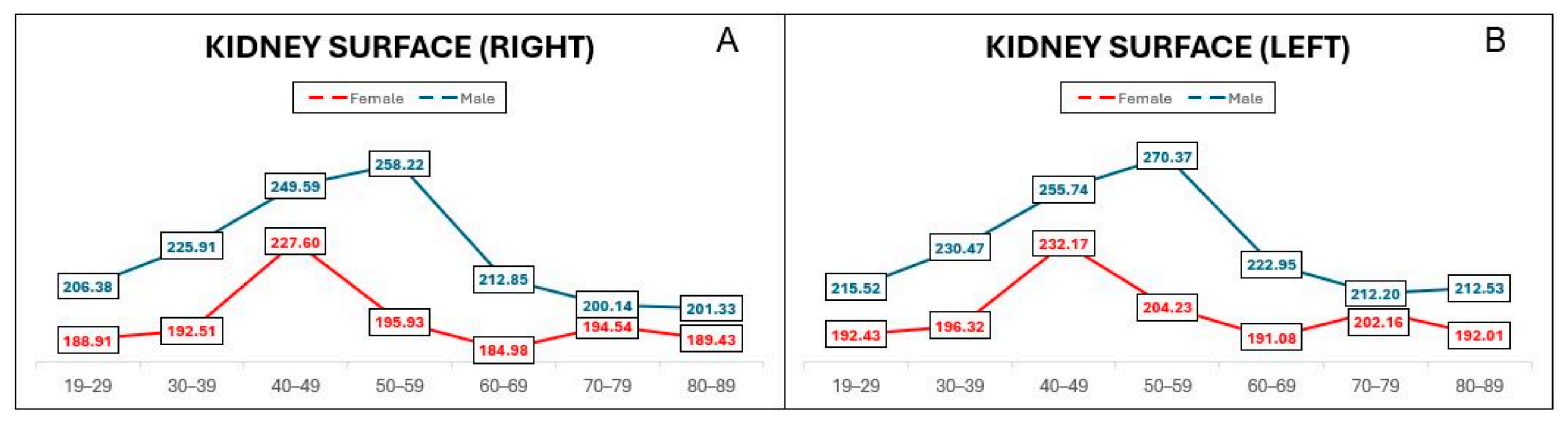
Change in right (**A**) and left (**B**) kidney area measurement by age group and sex.

As seen in Figure 6, the average kidney area decreases with age in both men and women, and it is observed that the average kidney area is higher in men than in women.

In conclusion, the volume and surface area findings largely support each other. In men, both parameters were higher than in women. A significant decline was observed after the age of 70. The left kidney was larger than the right kidney in terms of both volume and surface area. The sex difference disappeared in older groups (≥70 years). These results support the atrophy of the renal parenchyma, nephron loss, and decreased renal blood flow with aging.

## 4. Discussion

According to the findings of our study, the kidney volume and surface area values of men were found to be statistically significantly higher than those of women in almost all age groups. This difference was particularly noticeable in the 30–69 age range. This finding is completely parallel to the USG study conducted by Emamian and colleagues on 665 volunteers. Glodny et al.’s large-scale CT study of 1040 asymptomatic adults did not measure kidney volume, but the length and width of the kidney were measured, and the same intersex trends were reported. Both studies in the literature reported that kidney size was larger in men than in women [5,20,21].

The main reason for this sex difference is usually the higher body mass, muscle tissue, and consequently higher metabolic needs of the kidneys, which are generally higher in men. The kidneys grow in proportion to the body’s overall metabolic activity; therefore, it is physiologically expected that men with higher metabolic demands require a larger filtration surface [20,21]. The presence of a strong correlation between body surface area and kidney volume suggests that this relationship has both an anatomical and physiological basis. This study clearly demonstrates this difference by presenting absolute volume values.

Our study clearly shows that kidney volume and surface area decrease gradually with age in both sexes. The highest volume values in women were observed in the 40–49 age group, while in men, they were seen in the 30–59 age group, and a significant decline was recorded in both sexes after the age of 70. This age-related shrinking trend is consistent with findings reported in other studies, such as those by Gong and Emamian [5,21]. The underlying mechanism behind this regression is age-related physiological processes: renal parenchymal atrophy, progressive loss of nephron number, and decreased renal blood flow [22]. One of the most important findings is that the difference in kidney volume between women and men in the 70 and over age groups loses statistical significance. This suggests that the common loss of nephrons and fibrosis seen in later life has such a strong impact that it overshadows the initial sex differences. Aging leads to a similar pattern of kidney tissue shrinkage across sexes, reducing initial differences in kidney physiology [23].

In our study, the volume and surface area values of the left kidney were found to be statistically significantly higher than those of the right kidney. This finding is consistent with extensive literature data, including the ultrasonography-based study by Emamian et al. and the CT-based study by Shin et al. [3,21]. The primary anatomical explanation for the left kidney being larger than the right is that the liver is located in the right upper quadrant, pushing the right kidney upwards and causing it to take on a more compact form [21]. This anatomical difference suggests that the asymmetry in the size of the kidneys is a normal physiological variation. Therefore, it is necessary not to interpret this asymmetry as a pathological condition in clinical assessments. Our study reconfirms this anatomical reality using a different technological method.

In the study by Emamian and colleagues, the median volumes obtained using ultrasound and the ellipsoid formula (Right: 134 cm^3^, Left: 146 cm^3^) were lower than the average volumes we obtained with CT-based 3D segmentation (Right: 154.45 ± 34.06 cm^3^, Left: 157.73 ± 34.88 cm^3^). These quantitative differences are largely due to the nature of the measurement methods used. USG-based ellipsoid formulas tend to systematically underestimate true volume because they simplify the irregular shape of the kidney. Torres and colleagues also noted that USG has limitations in segmentation due to its low contrast and noise. In contrast, three-dimensional segmentation applied to CT data more accurately calculates the entire anatomical volume of the kidney [16]. This is a methodological superiority that explains why our study obtained higher volume values than Emamian’s study. This comparison highlights the importance of technological advances and accurate measurement methods in the assessment of kidney morphometry.

The 3D Slicer program and the Total Segmentator plugin used in our study offer a fast and reproducible automatic segmentation method. This approach allows for the analysis of large patient cohorts, as it requires less time and effort compared to traditional manual delineation. Current studies have reported that deep learning—based automatic segmentation algorithms show high correlation with manual segmentation and improve interobserver agreement [6,8]. This proves that the method used in our study is consistent with the latest technological trends in radiology.

The present study provides comprehensive CT-derived morphometric data on renal volume and surface area across different age groups and between sexes, offering additional information on physiological variations in renal morphology. These findings may facilitate the interpretation of renal morphometric measurements in a variety of clinical settings. For example, progressive reductions in kidney size are commonly observed in chronic kidney disease, and decreased renal volume has been associated with disease progression, nephron loss, and declining renal function [11,24]. Similarly, renal volume has been identified as an independent predictor of graft function and prognosis following living donor kidney transplantation [25,26]. Beyond volumetric assessment, advances in three-dimensional image analysis have expanded the clinical applications of platforms such as 3D Slicer, including preoperative planning, surgical simulation, and quantitative evaluation of renal calculi and other renal pathologies [13,14]. Collectively, these findings emphasize the growing clinical importance of quantitative renal morphometry in diagnostic evaluation, surgical planning, and patient management.

Although renal volume remains the most widely used and clinically validated three-dimensional morphometric parameter, renal surface area has recently gained attention as a complementary descriptor of renal morphology. Jones et al. reported that surface-related renal measurements were closely associated with renal mass, suggesting that these measurements may provide additional structural information [27]. More recently, Lee et al. evaluated renal volume, surface area, and surface area-to-volume ratio using CT-based three-dimensional morphometric analysis and demonstrated that surface-related parameters may provide complementary information regarding structural renal alterations [28]. In the present study, renal surface area was strongly correlated with renal volume (r = 0.88), indicating that both parameters largely reflect overall kidney size. Nevertheless, because they characterize different morphological properties, we considered it appropriate to report both measurements to provide a more comprehensive three-dimensional assessment of renal morphology. Although renal volume currently has broader clinical validation, the independent clinical utility of renal surface area requires further investigation in studies incorporating renal functional parameters and clinical outcomes.

This study has several limitations. First, its retrospective single-center design may limit the generalizability of the findings, as the study population was derived from a single institution in Türkiye and may not fully represent populations with different ethnic backgrounds, referral patterns, or imaging protocols. Second, renal morphometric measurements were not normalized for anthropometric variables such as height, weight, body mass index (BMI), and body surface area (BSA), which may have influenced the observed age- and sex-related differences. Third, biochemical indicators of renal function, including serum creatinine and estimated glomerular filtration rate (eGFR), were not available; therefore, the relationship between renal morphometry and kidney function could not be assessed, and subclinical renal dysfunction cannot be completely excluded despite the exclusion of individuals with documented hypertension, diabetes mellitus, chronic kidney disease, and radiologically detectable renal abnormalities. Future multicenter prospective studies involving ethnically diverse populations and incorporating anthropometric measurements, renal functional parameters, and clinical outcome data are warranted to further validate and extend these findings.

## 5. Conclusions

In this study, kidney volume and surface area measurements were performed using 3D Slicer software and evaluated according to age and sex groups. Our findings indicate that the left kidney is larger than the right kidney and that renal size peaks in middle age, followed by a gradual decline with advancing age. However, the disappearance of sex differences after the age of 70 is consistent with age-related renal atrophy. Our results support the data reported in the literature and demonstrate that the 3D Slicer program provides a reliable assessment of kidney morphometry.

## Figures and Tables

**Table 1:** Distribution of age groups by sex.

	Sex		Total (*n* = 291)	*χ* ^2^	*p*
	Female (*n* = 145)	Male (*n* = 146)			
	*n* (%)	*n* (%)	*n* (%)		
Age (years)				0.232	0.999
19–29	20 (14%)	19 (13%)	39 (13%)		
30–39	23 (16%)	24 (17%)	47 (16%)		
40–49	26 (18%)	26 (18%)	52 (18%)		
50–59	17 (11%)	19 (13%)	36 (12%)		
60–69	20 (14%)	21 (14%)	41 (14%)		
70–79	20 (14%)	19 (13%)	39 (14%)		
80–89	19 (13%)	18 (12%)	37 (13%)		

**Table 2:** Comparison of right and left kidney volume measurements by sex in age groups.

	Age (Years)	Right	Left	Mean Paired Difference, Left–Right (95% CI), cm^3^	*t* *	*p*	*Cohen’s dz*
		*X* ± *SD*	*X* ± *SD*				
**Female**	19–29	144.30 ± 27.99	150.90 ± 33.27	6.60 (0.11 to 13.09)	2.129	0.047 *	0.48
	30–39	149.98 ± 33.41	154.21 ± 32.31	4.24 (−4.11 to 12.58)	1.052	0.304	0.22
	40–49	156.78 ± 37.51	157.15 ± 33.23	0.37 (−7.58 to 8.33)	0.096	0.924	0.02
	50–59	144.36 ± 26.45	152.80 ± 29.50	8.44 (0.34 to 16.55)	2.208	0.042 *	0.54
	60–69	134.05 ± 21.88	136.79 ± 23.10	2.74 (−3.17 to 8.66)	0.970	0.344	0.22
	70–79	141.60 ± 26.74	147.12 ± 38.85	5.52 (−8.76 to 19.81)	0.809	0.429	0.18
	80–89	120.34 ± 23.58	120.82 ± 25.42	0.48 (−6.09 to 7.04)	0.153	0.880	0.04
	All Ages	142.52 ± 30.71	146.36 ± 32.89	3.84 (0.76 to 6.92)	2.465	0.015 *	0.20
**Male**	19–29	161.08 ± 17.94	167.28 ± 19.10	6.20 (2.17 to 10.23)	3.235	0.005 *	0.74
	30–39	182.85 ± 26.42	183.03 ± 33.16	0.17 (−7.50 to 7.84)	0.047	0.963	0.01
	40–49	176.05 ± 27.38	177.92 ± 25.41	1.87 (−5.94 to 9.67)	0.493	0.626	0.10
	50–59	184.75 ± 38.85	188.79 ± 33.06	4.04 (−7.29 to 15.37)	0.749	0.464	0.17
	60–69	161.81 ± 33.36	164.13 ± 25.53	2.32 (−12.19 to 16.83)	0.334	0.742	0.07
	70–79	142.79 ± 26.83	159.87 ± 38.56	17.08 (−1.84 to 35.99)	1.897	0.074	0.44
	80–89	141.37 ± 34.28	143.82 ± 40.93	2.45 (−9.11 to 14.01)	0.447	0.661	0.11
	All Ages	165.70 ± 33.33	170.25 ± 33.58	4.55 (0.49 to 8.61)	2.217	0.028 *	0.18
Total		154.15 ± 34.05	158.35 ± 35.27	4.20 (1.66 to 6.73)	3.259	0.001 *	0.19

* *p* < 0.05. Right- and left-kidney volumes were compared using the paired-samples *t*-test. Mean paired differences and corresponding *t* values were calculated as left minus right kidney volume; therefore, positive values indicate a greater left-kidney volume. Cohen’s dz was reported as the standardized effect size for paired comparisons. CI, confidence interval; SD, standard deviation.

**Table 3:** Comparison of right and left kidney surface area measurements by sex in age groups.

	Age (Years)	Right	Left	Mean Paired Difference, Left–Right (95% CI), cm^2^	t *	*p*	Cohen’s dz
		*X* ± *SD*	*X* ± *SD*				
**Female**	19–29	206.38 ± 17.28	215.52 ± 22.64	3.51 (−3.58 to 10.61)	2.916	0.009 *	0.23
	30–39	192.51 ± 27.18	196.32 ± 30.45	3.81 (−4.05 to 11.66)	1.005	0.326	0.21
	40–49	227.60 ± 37.23	232.17 ± 35.86	4.56 (−5.20 to 14.33)	0.963	0.345	0.19
	50–59	195.93 ± 24.44	204.23 ± 26.07	8.30 (−2.04 to 18.65)	1.701	0.108	0.41
	60–69	184.98 ± 23.33	191.08 ± 24.33	6.10 (−0.65 to 12.85)	1.891	0.074	0.42
	70–79	200.14 ± 23.99	212.20 ± 29.83	7.62 (−5.35 to 20.59)	2.869	0.010 *	0.28
	80–89	189.43 ± 26.69	192.01 ± 33.36	2.58 (−7.49 to 12.65)	0.539	0.597	0.12
	All Ages	197.55 ± 30.98	202.66 ± 34.67	5.11 (1.77 to 8.45)	3.024	0.003 *	0.25
**Male**	19–29	210.07 ± 19.58	212.89 ± 18.98	9.14 (2.55 to 15.72)	2.067	0.053	0.67
	30–39	225.91 ± 25.98	230.48 ± 32.64	4.57 (−1.49 to 10.63)	1.559	0.133	0.32
	40–49	249.59 ± 28.94	255.74 ± 30.13	6.15 (−2.70 to 15.00)	1.432	0.164	0.28
	50–59	258.22 ± 38.06	270.37 ± 34.08	12.15 (−0.34 to 24.65)	2.044	0.056	0.47
	60–69	212.85 ± 37.60	222.95 ± 27.22	10.11 (−4.91 to 25.12)	1.404	0.176	0.31
	70–79	202.77 ± 23.69	210.10 ± 27.82	12.06 (3.23 to 20.90)	2.013	0.059	0.66
	80–89	201.33 ± 35.82	212.53 ± 45.31	11.20 (−4.23 to 26.63)	1.531	0.144	0.36
	All Ages	223.53 ± 36.79	232.55 ± 37.77	9.02 (5.24 to 12.80)	4.714	<0.001 *	0.39
Total		210.58 ± 36.37	217.65 ± 39.17	7.07 (4.55 to 9.59)	5.526	<0.001 *	0.32

* *p* < 0.05. Right- and left-kidney surface areas were compared using the paired-samples *t*-test. Mean paired differences were calculated as left minus right kidney surface area; therefore, positive values indicate a greater left-kidney surface area. Cohen’s dz was reported as the standardized effect size for paired comparisons. CI, confidence interval; SD, standard deviation.

**Table 4:** Comparison of right and left kidney volume measurements in age groups by sex.

Volume		Female	Male		
		*X* ± *SS*	*X* ± *SS*	*t*	*p*
Right	19–29 years	144.65 ± 28.27 ^ab^	164.24 ± 18.26 ^ab^	−2.555	0.015 *
	30–39 years	149.98 ± 33.41 ^a^	182.85 ± 26.42 ^a^	−3.750	0.001 *
	40–49 years	156.78 ± 37.51 ^a^	176.05 ± 27.38 ^a^	−2.116	0.039 *
	50–59 years	145.54 ± 25.69 ^ab^	184.75 ± 38.85 ^a^	−3.527	0.001 *
	60–69 years	134.05 ± 21.88 ^ab^	161.81 ± 33.36 ^ab^	−3.134	0.003 *
	70–79 years	141.60 ± 26.74 ^ab^	142.79 ± 26.83 ^b^	−0.138	0.891
	80–89 years	120.34 ± 23.58 ^b^	141.37 ± 34.28 ^b^	−2.185	0.036 *
Test Statistics	*F*	3.394	7.178		
	*p*	0.004 *	<0.001 *		
	*η* ^2^	0.129	0.237		
Left	19–29 years	150.90 ± 33.27 ^ab^	167.28 ± 19.10 ^ab^	−1.872	0.069
	30–39 years	154.21 ± 32.31 ^a^	183.03 ± 33.16 ^a^	−3.015	0.004 *
	40–49 years	157.15 ± 33.23 ^a^	177.92 ± 25.41 ^a^	−2.531	0.015 *
	50–59 years	152.80 ± 29.50 ^a^	188.79 ± 33.06 ^a^	−3.429	0.002 *
	60–69 years	136.79 ± 23.10 ^ab^	164.13 ± 25.53 ^ab^	−3.590	0.001 *
	70–79 years	147.12 ± 38.85 ^ab^	159.87 ± 38.56 ^ab^	−1.028	0.311
	80–89 years	120.82 ± 25.42 ^b^	143.82 ± 40.93 ^b^	−2.066	0.046 *
Test Statistics	*F*	**3.350**	**4.695**		
	*p*	0.004 *	<0.001 *		
	*η* ^2^	**0.127**	**0.169**		

* *p* < 0.05. Independent-samples *t*-test was used for comparisons between females and males within each age group. One-way analysis of variance was used to compare age groups separately within each sex. Within the same sex column, means that do not share a common superscript letter differ significantly according to the Tukey HSD post-hoc test (*p* < 0.05). Descriptive statistics are presented as mean ± standard deviation. η^2^ indicates eta-squared effect size.

**Table 5:** Comparison of right and left kidney area measurements in age groups by sex.

Surface		Female	Male		
		*X* ± *SS*	*X* ± *SS*	*t*	*p*
Right	19–29 years	188.91 ± 22.66 ^b^	206.38 ± 17.28 ^c^	−2.697	0.010 *
	30–39 years	192.51 ± 27.18 ^b^	225.91 ± 25.98 ^bc^	−4.307	0.000 *
	40–49 years	227.60 ± 37.23 ^a^	249.59 ± 28.94 ^ab^	−2.377	0.021 *
	50–59 years	195.93 ± 24.44 ^b^	258.22 ± 38.06 ^a^	−5.764	0.000 *
	60–69 years	184.98 ± 23.33 ^b^	212.85 ± 37.60 ^c^	−2.834	0.007 *
	70–79 years	194.54 ± 27.74 ^b^	200.14 ± 23.99 ^c^	−0.673	0.505
	80–89 years	189.43 ± 26.69 ^b^	201.33 ± 35.82 ^c^	−1.150	0.258
Test Statistics	*F*	6.431	12.255		
	*p*	<0.001 *	<0.001 *		
	*η* ^2^	0.219	0.346		
Left	19–29 years	192.43 ± 29.07 ^b^	**215.52 ± 22.64** ^c^	−2.757	0.009 *
	30–39 years	196.32 ± 30.45 ^b^	230.47 ± 32.64 ^bc^	−3.706	0.001 *
	40–49 years	232.17 ± 35.86 ^a^	255.74 ± 30.13 ^ab^	−2.566	0.013 *
	50–59 years	204.23 ± 26.07 ^ab^	270.37 ± 34.08 ^a^	−6.480	0.000 *
	60–69 years	191.08 ± 24.33 ^b^	222.95 ± 27.22 ^c^	−3.945	0.000 *
	70–79 years	202.16 ± 40.87 ^b^	**212.20 ± 29.83** ^c^	−0.872	0.389
	80–89 years	192.01 ± 33.35 ^b^	212.53 ± 45.31 ^c^	−1.575	0.124
Test Statistics	*F*	4.929	10.329		
	*p*	<0.001 *	<0.001 *		
	*η* ^2^	0.176	0.308		

* *p* < 0.05. Independent-samples *t*-test was used to compare females and males within each age group. One-way analysis of variance was used to compare age groups separately within each sex. Within the same sex column, means that do not share a common superscript letter differ significantly according to the Tukey HSD post-hoc test (*p* < 0.05). Descriptive statistics are presented as mean ± standard deviation. η^2^ indicates the eta-squared effect size.

## Data Availability

The data supporting the findings of this study are available from the corresponding author upon reasonable request.

## Supplementary Materials

The following supporting information can be downloaded at: https://www.mdpi.com/article/10.3390/life16081347/s1. Table S1. Significant Tukey HSD pairwise comparisons of kidney volume across age groups by sex. Table S2. Significant Tukey HSD pairwise comparisons of kidney surface area across age groups by sex.
